# Regulating health professional scopes of practice: comparing institutional arrangements and approaches in the US, Canada, Australia and the UK

**DOI:** 10.1186/s12960-020-00550-3

**Published:** 2021-01-28

**Authors:** Kathleen Leslie, Jean Moore, Chris Robertson, Douglas Bilton, Kristine Hirschkorn, Margaret H. Langelier, Ivy Lynn Bourgeault

**Affiliations:** 1 Athabasca University and Co-Lead, Regulation and Governance Theme, Canadian Health Workforce Network, Athabasca, Canada; 2 Center for Health Workforce Studies, School of Public Health, University at Albany, State University of New York, Rensselaer, NY USA; 3 Australian Health Practitioner Regulation Agency, Melbourne, Australia; 4 Standards and Policy, Professional Standards Authority, London, United Kingdom; 5 Government of Canada, Ottawa, Canada; 6 University of Ottawa and Lead, Canadian Health Workforce Network, Ottawa, Canada

**Keywords:** Professional regulation, Scopes of practice, Health professions, US, Canada, UK, Australia

## Abstract

**Background:**

Fundamentally, the goal of health professional regulatory regimes is to ensure the highest quality of care to the public. Part of that task is to control what health professionals do, or their *scope of practice*. Ideally, this involves the application of evidence-based professional standards of practice to the tasks for which health professional have received training. There are different jurisdictional approaches to achieving these goals.

**Methods:**

Using a comparative case study approach and similar systems policy analysis design, we present and discuss four different regulatory approaches from the US, Canada, Australia and the UK. For each case, we highlight the jurisdictional differences in how these countries regulate health professional scopes of practice in the interest of the public. Our comparative Strengths, Weaknesses, Opportunities, Threats (SWOT) analysis is based on archival research carried out by the authors wherein we describe the evolution of the institutional arrangements for form of regulatory approach, with specific reference to scope of practice.

**Results/conclusions:**

Our comparative examination finds that the different regulatory approaches in these countries have emerged in response to similar challenges. In some cases, ‘tasks’ or ‘activities’ are the basis of regulation, whereas in other contexts protected ‘titles’ are regulated, and in some cases both. From our results and the jurisdiction-specific SWOT analyses, we have conceptualized a synthesized table of leading practices related to regulating scopes of practice mapped to specific regulatory principles. We discuss the implications for how these different approaches achieve positive outcomes for the public, but also for health professionals and the system more broadly in terms of workforce optimization.

## Background

Fundamentally, the goal of health professional regulatory regimes is to ensure the highest quality of care to the public. Part of that task is to monitor and control what health professionals do, or their scope of practice. Ideally, this involves the application of evidence-based professional standards of practice to the tasks for which health professionals have received training. There are different regulatory approaches to achieving these goals across international and regional jurisdictions.

Regulatory authority lies in a range of professional as well as state institutions, revealing a continuum from professional autonomy to state control [1]. That is, professions and governments share regulatory authority to varying degrees, historically and between countries. There is even further complexity in the more contemporary era because in many cases new agencies and partnership organizations have been created to regulate at arms’ length from the professions and the state, in some cases in coordination across health professions within a given workforce. A shift from traditional professional self-regulation has occurred in many jurisdictions, often as a result of regulatory failures to protect the public [2]. Moreover, the regulation of health professionals and their work has become more constrained as governments strive to provide not only high-quality, but also cost-effective care to the public.

In this paper, we present and discuss four different regulatory approaches from the United States (US), Canada, Australia, and the United Kingdom (UK), highlighting the differences in how these countries regulate health professional scope of practice in the interest of the public. Across countries, the aims may be similar, but the mechanisms differ because of local historical policy legacies and cultural norms [2]. This makes comparative work challenging but also interesting. Whereas previous analyses have focused on professions and strategies, we focus on scopes of practice and institutional approaches. Our comparative examination finds that the different regulatory approaches in these countries have different implications for how health professional scopes are articulated and in turn how this can impact on health workforce optimization. They have each experienced different forms of regulatory failure and each are undertaking, in some cases, substantial reforms of their health professional regulatory models partly in response to these failures.

In this article, we are focused on the system-level impact of health professional regulation on scopes of practice. Regulation is only one of many system variables that influence scopes of practice. Others include collaborative practice agreements, delegation and supervision models, board certification, and funding arrangements. Professional associations may also impact scope of practice by seeking to defend or extend their members’ current scope as part of their advocacy role [3]. With few exceptions, however, the role of the regulator is separate from the role of professional associations and the regulators’ mandate of protecting the public interest imposes a unique impact on scope of practice.

Currently, one of the major influences on health professional scopes of practice globally is the COVID-19 pandemic. The pandemic has made clear the necessity of optimizing the workforce by ensuring all professionals are practicing to full scope [4]. Flexibility in scope has also been emphasized as a means to augment the health workforce, particularly in specific pandemic response areas. Maintaining public protection while ensuring access to the needed workforce is an important aspect of regulating scopes of practice that has become even more critical during the current public health crisis.

## Health profession regulation, public protection, and scopes of practice

Although the core concern of health profession regulation is protection of the public, historically, achieving self-regulatory status was considered akin to achieving professional status [5, 6]. As Larson [7] describes, regulation often helped to create a state-sponsored monopoly over the profession’s services. This status attainment may have been afforded to only a few professions, reflecting a hierarchy within a division of labour. Health professional regulation, for example, often resulted in the constraining of a health professions’ scope of practice or modality ‘crystalizing’ the dominance of the medical profession [8, 9]. This differential outcome speaks to the dynamic relations that exist between the state and professions; a relationship that has evolved from granting state-sanctioned self-regulatory status [10] to funding health professional services and broader systems management.

Contemporary approaches to health professional regulation have increasingly focused on improving accountability to the public interest through more open, transparent, and publicly accountable processes for peer surveillance and control, in light of clinical mistakes threatening the safety of patient care [11–15]. Regulation by design should protect patients from the possibly deleterious effects of asymmetrical information between them and health professionals [16]. A focus on ‘patient safety’ challenges, what was traditionally the technical and esoteric domains of health professionals, was increasingly brought into the scope of political and managerial reform in healthcare [17].

The principles of *right-touch regulation*—where the level of regulation is proportionate to the level of risk to the public—have been used in varied ways by regulators across international jurisdictions to modernize regulation in the public interest [18]. This focus on risk management also highlights how health professions regulation must be flexible enough to support efficient and effective use of the health workforce. Nelson et al. [19] argued that regulating scopes of practice requires balancing the intersecting dimensions of *flexibility*—empowering teams to determine the relative responsibilities of the different practitioners based upon community need; and *accountability*—ensuring the optimization of scopes of practice within a professional regulatory environment. This intersection has sharpened during the COVID-19 pandemic with regulators being urged to enhance their flexibility, while maintaining accountability for protecting the public, as a way to augment the health workforce [20].

The pandemic has also highlighted a fundamental truth: society moves quickly while regulation and law tend to be more static, and regulatory frameworks need to be made nimbler and more responsive to meet the needs of modern society [21]. This is true as well in the regulation of scopes of practice as modern team-based care and technological advances increasingly transform health professional work. Despite the nexus between modernizing regulation and optimizing the health workforce, there is a gap in knowledge around the impact of different regulatory models on health professional scopes of practice.

## Methods

We employed a comparative case study approach, informed by Yin’s [22] case study methodology, utilizing a similar systems policy analysis design [23]. This involved the collection and analysis of policy documents, published, and grey literature by locally situated investigators [24]. In two cases (US and Canada), the investigators were situated at arms-length from a professional regulatory body. In the other two cases (UK and Australia), the investigators are situated within regulatory structures. A common analytic template guided the comparative analysis along key institutional dimensions. Refinement of a preliminary analysis was undertaken after presentation to two different international audiences [25, 26].

The unit of comparative analysis is at the systems-level rather than the individual profession level, with the comparison focused across the regulatory approaches in each jurisdiction. For each case, the health professions regulatory framework used in each country is described highlighting the emergence of new institutional structures between professions and the government (state) and approaches to regulating health professional scopes of practice. The presentation of the data is both thematic and semi-chronological by country, highlighting key events that have shifted structures, organizations, and interests. We draw out of each case the strengths, weaknesses, opportunities, and threats (SWOT) institutional analysis which enables an appreciation of the implications of the different regulatory approaches for health professional scopes of practice and health workforce optimization. From our results and these SWOT analyses, we have conceptualized a synthesized table of leading practices related to regulating scopes of practice mapped to specific regulatory principles.

## Results

### The United States: regulating strict scopes of practice

In the United States, the regulation of health professions primarily falls to the states. State-based laws and regulations define specific legal scopes of practice for health professionals including the health services that can be legally offered (e.g., controlled acts) and the circumstances under which these services may be provided (the context for professional practice). Regulation occurs under the auspices of a range of state agencies, including departments of health, education, and other state agencies in which regulatory boards are housed. The configurations of state boards vary in scope of authority, level of autonomy, and control over administrative processes [27]. A 2020 report on state regulatory structures provides a comprehensive overview of these configurations and included survey responses from 161 representatives in 45 states and the District of Columbia [27]. In 26 of these jurisdictions, regulatory boards had full autonomy in decision-making; 16 states employed a mixed model where some regulatory bodies had autonomy while others used a central agency for decision-making. Four states relied exclusively on a central agency for decision-making and boards functioned only in an advisory capacity.

Although states have the constitutional authority to govern regulatory processes, the federal government is able to influence state governance. Recent advisory opinions from the Federal Trade Commission and several court rulings, including one from the US Supreme Court, cite the inherent risk in self-regulation, i.e. the potential for professional self-protection [28] and monopolistic practice in violation of federal anti-trust laws [29]. These advisories recommend active oversight of certain board decisions by external bodies [30] to avoid unnecessarily anti-competitive outcomes. Several states have proposed changes in the structure of professional regulatory boards or have created centralized review boards with the power to accept or reject board recommended regulatory changes [29, 30].

A critical challenge associated with the regulation of health professions is state-to-state variation in scope of practice, which is limited by the location of the professional rather than by their skills and competencies. Most health professionals in the US are trained in nationally accredited educational programmes using standard curriculum and most complete national competency exams. Despite these national standards, some states limit a health professional’s ability to practice to the full scope of their demonstrated professional competency. State-based laws can restrict practice, which is especially relevant in times of crisis, and can also impede the provision of health services across state boundaries [31].

State licensing laws may also impede services when the consulting clinician is licensed in a state other than the one where the patient is located. To address this barrier, some states have opted to join *interstate licensure compacts* that allow a clinician who meets licensure requirements in one state to practice in other states in the compact. Although the models differ in detail, there is now a nurse licensure compact effective in 25 states [32] and an interstate medical licensure compact effective in 29 states and DC [33]. Other professions are developing interstate compacts as well, including emergency medical personnel and physical therapy. These compacts have been especially useful during the COVID-19 outbreak in the US, allowing clinicians to cross state borders and practice where there was great demand for health workers. In addition, in response to the need to quickly build workforce surge capacity during the pandemic, some states issued emergency regulations allowing physicians, nurses and other health professionals licensed and in good standing in other states to practice in their state.

Efforts to recognize new professions or modify scope of practice for existing health professions usually require the enactment of or amendment to state law, a process which is typically slow and, at times, adversarial. States often solicit input on proposed changes from stakeholders, including professional associations and, to a more limited extent, consumer groups. Emerging professions with fewer resources to mount advocacy campaigns may be disadvantaged in this process by more powerful and well-funded professional constituencies in a state.

Scope of practice regulations also affect the distribution of health professionals. Supervision requirements often result in the co-location of NPs and PAs with physicians, which limits their dispersion in underserved areas and results in reduced primary care capacity, especially in rural locations. A 2018 study that examined supply and distribution of NPs in the US found that the supply of NPs in geographic areas designated as health professions shortage areas was highest in states that recognized more autonomous scopes of practice for NPs [34]. In response to the COVID pandemic, some states temporarily waived supervisory requirements for NPs and PAs, which enabled them to practice where needed [35].

Health care in the US is changing and these changes have heightened discussion about the impacts of scopes of practice on access to needed services. Enacting regulations to support overlapping scopes of practice among health professionals is at the crux of many of the contentious debates occurring in states. Standardizing scopes of practice for health professions based on competencies would enable service delivery unencumbered by state boundaries. Proposing the establishment of new professions and expanding practice for existing professions must be based on the best available evidence and be within the parameters of training and competency for the profession [36].

Table 1 details the SWOT analysis of the US case.

**Table 1:** The SWOT analysis of the US case

**Strengths** Supports workforce innovation responsive to local needs Licensure compacts allowing licensure recognition and sharing of regulatory data across jurisdictions	**Weaknesses** Patchwork of approaches and reforms across jurisdictions Process for changing state regulations is slow, adversarial and costly Failure to reconcile legal scope of practice with professional competency in state regulation
**Opportunities** Increasing use of interprofessional team-based models of care Enables the ‘testing’ of innovative models in one state for potential implantation across other states Payment reform Focus on population health	**Threats** Restrictive scopes of practice are inefficient and can limit access to care Failure to systematically evaluate workforce innovation

### Canada: regulating flexible scopes of practice through tasks

Health professional regulation falls under provincial and territorial jurisdiction in Canada. As such, and despite national-level accreditation and educational standards for many professions, there is substantial variation across the country in terms of regulatory models, which professions are regulated, and the activities that are regulated. As in the US case, this results in differential access to providers and services across the country.

What is common across Canada is the self-regulatory status of most health professions via the statutory delegation of authorities to the ministers of health to establish regulations, and to regulatory authorities (often called regulatory colleges) to govern their respective professions [37–39]. The introduction of new regulated health professions and scopes of practice changes therefore require either legislative or regulatory amendment. Professional regulatory authorities are responsible for establishing entry-to-practice credentials, maintaining a public register of health professionals, upholding standards of practice, and overseeing complaints and disciplinary proceedings. The regulators are expected to act in the public interest, which distinguishes them from professional associations that focus on professional interests [38–42].

The traditional model of health profession regulation across Canadian provinces is based on separate statutes and exclusive scopes of practice for each profession. There has been a trend to move away from this model towards umbrella frameworks characterized by overlapping scopes of practice [38, 39, 43–53]. This began with the *Regulated Health Professions Act*, *1991* (RHPA) [54] in Ontario and other provinces have since followed with similar umbrella legislation.

Umbrella frameworks apply uniform standards to the health professions that are governed by the legislation. The legislation sets out consistent provisions for governance, registration, complaints, discipline, appeals, public representation, regulation and by-law making powers. The umbrella act is accompanied by specific regulations or statutes for individual professions that confer title protection and include broad, non-exclusive scope of practice statements. These legislative statements are then used by the regulatory bodies to develop competencies, guidelines, and standards of practice. Legislative scope of practice statements and regulatory policies generally set the outer limits of the professions’ scope of practice.

In addition to title protection and non-exclusive scope of practice statements, the umbrella legislative frameworks enumerate a number of controlled or restricted acts. These controlled or restricted acts are an effort to balance promoting interdisciplinary care while still restricting higher risk activities to specific professional groups [39]. The same controlled activities may be granted to more than one profession and may also be delegated. The dominant position physicians maintain in the health care system is reflected in how many controlled acts they are authorized to perform [55].

The introduction of overlapping scopes of practice through the non-exclusive scope of practice statements and controlled acts model is also intended to enhance flexibility in the provider(s) who deliver services, as well as encourage interprofessional practice [47–49]. Other statutory changes to encourage collaboration, team-based models of care, and new providers have accompanied or followed the introduction of these umbrella frameworks [45, 50–52]. Newfoundland and Labrador, for example, has established the Council of Health Professionals, an independent body that is responsible for coordinating the regulation of eight health professions. In Ontario, 2009′s *Regulated Health Professions Statute Law Amendment Act* [56] mandated the health regulators collaborate in the development of standards where controlled acts are overlapping.

Umbrella legislation with overlapping scopes of practice does not entirely prevent scopes of practice from being a barrier to collaborative, team-based care, as it effectively entrenches a narrower range of controlled activities [50]. A 2018 report commissioned by the Ontario government identified the current system as ill-suited for the future since it prevents a health professional from embracing a broader scope of practice or engaging in a controlled act even if that professional can demonstrate an appropriate level of competence [57]. These umbrella frameworks have nonetheless been considered a key instrument for introducing regulatory flexibility and loosening the restrictiveness of scopes [44, 50]. At the very least, “Umbrella legislation with more flexible scopes of practice provides a possible foundation for collaborative models of care” ([19], p. 54).

Another Canadian province, Nova Scotia, has taken an alternative approach to facilitating regulatory collaboration and flexible scopes of practice. In 2012, Nova Scotia introduced the *Regulated Health Professions Network Act* [58] to establish a statutory Network of self-regulating health professions that enables voluntary regulatory collaboration. The Network legislation authorizes regulatory authorities to enter into agreements respecting the interpretation or modification of scopes of practice without the need for further legislative amendment, provided the provincial health minister determines the agreement is in the public interest [59]. Reform is currently proposed in British Columbia that would see a reduction in the number of regulatory authorities from 20 to six [60]; this reform recognizes that regulating single professions in isolation does not allow regulatory colleges to respond nimbly to the complexities of modern team-based care.

Health professional scopes of practice in Canada have traditionally been enshrined in regulatory regimes on the basis of history and politics rather than best utilizing skills and knowledge best meet contemporary population health needs [19]. That seems to be changing with the complement of regulatory reform recently undertaken and currently proposed in Canada to facilitate collaboration and provide more flexibility in order to support health workforce innovations. The COVID-19 pandemic has necessitated further emergency reforms to facilitate surge capacity, such as policies to allow for internationally educated health professionals to be granted emergency licensure and upskilling to allow for pandemic-related task shifting [61]. However, the continued reliance by most Canadian provinces on discrete regulatory authorities for individual professions and the lack of national coordination around scopes of practice form barriers to interjurisdictional mobility and efficient health workforce reform.

Table 2 details the SWOT analysis of the Canadian case.

**Table 2:** The SWOT analysis of the Canadian case

**Strengths** Supports workforce innovation responsive to local needs Umbrella frameworks offer flexibility through overlapping scopes of practice	**Weaknesses** Patchwork of approaches and reforms across jurisdictions Little addresses the structural embeddedness of medical dominance in health professional regulation
**Opportunities** Enables the ‘testing’ of innovative models in one province/territory for potential implantation across other jurisdictions Reforms emphasize interprofessional collaboration	**Threats** Lack of a national mechanism for scale up Regulation of expanded scopes tend to be restrictive and inefficient which can limit access to care

### Australia: national consistency in outer boundaries of scope of practice

Australia has seen a major transformation to the legal framework and institutions governing the regulation of health practitioners. Historically, the health professions were regulated by statute in models that were primarily profession specific and were based on both restricting practices and titles. This had the impact of constraining or protecting scopes of practice through statute and being relatively unresponsive to the changing needs of the population and design of the health care system and workforce. The legislation and institutions regulating health practitioners were separated not only by profession but also replicated for each of eight jurisdictions in the federation. This was a highly fragmented and duplicative system for a country relatively large by land mass yet with a small and distributed population. Despite having mutual recognition mechanisms, this model presented mobility barriers, different professional standards, and regulatory costs to practicing across borders.

In 2010, Australia moved away from this state/territorial regulatory system to the National Registration and Accreditation Scheme. This scheme was established with the enactment of the National Law (beginning with the *Health Practitioner Regulation National Law Act* [62]*,* enacted in Queensland as the host jurisdiction, followed by uniform legislation in the other states and territories). The National Law covers 15 registered professions. One regulatory agency, Ahpra, manages this scheme while 15 National profession-specific Boards establish professional standards for registration and practise for their respective professions. The National Boards are responsible for registering professionals, imposing any necessary conditions on registration, developing standards and codes of conduct, and considering complaints about registrants.

Scope of practice regulation in Australia is now primarily accomplished through title protection under the National Law and minimal scope of practice restrictions by the National Boards. The National Boards develop registration standards about the scope of practice of registered health practitioners and also address roles and competencies in broad terms in documents such as practice standards and codes of professional conduct, where there is often a requirement that the health professional recognize and work within the limits of their competence and scope of practice. For example, the Nursing and Midwifery Board of Australia includes a requirement that nurses practice within their scope of practice. The regulator defines scope of practice as “that in which nurses are educated, competent to perform, and permitted by law” and adds that “actual scope of practice is influenced by the context in which the nurse practices, the health needs of people, the level of competence and confidence of the nurse and the policy requirements of the service provider” (63), p. 6). As such, the National Boards do not provide detailed explanations of scope of practice or regulate through restricted acts but rather maintain the outer boundaries of practice through their registration and practice standards.

The National Boards are also authorized under the National Law to “endorse” the registration of certain professionals. An endorsement recognizes that a health professional has an extended scope of practice in a particular area of practice because they have additional qualifications that are approved by the National Board [64]. Registered nurses may be endorsed as nurse practitioners, for example, and dentists may be endorsed for the approved area of practice of conscious sedation. Each National Board sets the requirements for endorsement for areas of practice within their profession. These endorsements thus provide an expanded scope built upon the foundation and inclusive of the professional’s original scope of practice on registration in the profession.

In addition to endorsements, specialties and specialist titles may also be developed by the National Boards. As of the time of writing, specialist registrations may be granted in dentistry, medicine, and podiatry. A ministerial council comprised of the health ministers of all eight state and territories and the Commonwealth approves the standards set by each National Board for entry to practice, for endorsement of registration for advanced practice, and for specialist registration [65].

While the factors leading to endorsement and specialist registration by a National Board are generally straightforward, the expression of these expanded scope of practice is often shaped by federal, state, and territory legislation around funding and prescription drugs that can form a barrier to practicing to full scope [66–68]. This has been found in particular in relation to nurse practitioner scope of practice where jurisdiction and clinical context continues to be a major influence on defining scope of practice [66, 67]. Further, employers or professional associations may develop their own lists of skill sets that define scope of practice within specific settings or may use credentialing to verify the ability of practitioners to provide specialized practice within specific organizational environments [69].

As such, there remain jurisdictional differences in scopes of practice for some Australian health professions due to state legislation, clinical context-specific guidelines, and employer or professional association-level credentialing. However, the uniform legislation and regulatory authorities operating nationally provide title protection and set the outer boundaries of practice through registration and practice standards. This national coordination has been used during the COVID-19 pandemic response to facilitate a short-term sub-register for fast tracking a return to the workforce for experienced and qualified professionals [70] and to provide national guidance on telehealth [71].

Table 3 details the SWOT analysis of the Australian case.

**Table 3:** The SWOT analysis of the Australian case

**Strengths** Uniform legislation and regulatory authorities operating nationally provide consistent and clear practice standards and regulatory frameworks Mobility of practitioners across internal borders Flexible scope of practice to enable and respond to changing models of care Increased public engagement through community reference group	**Weaknesses** Complex internal arrangements Conservative bias to regulation Professional silos transferred into new model
**Opportunities** Reforms emphasize interprofessional collaboration Focus on evidence-informed risk-based regulation Focus on greater inter-professional practice National, standardized data fosters health workforce planning	**Threats** Delivering successful cultural change required to be effective, contemporary, and relevant Bigger political target

### The United Kingdom: differing regulatory approaches in a complex landscape

In the UK, there are ten separate statutory organizations, mostly called councils, that regulate health professionals. These ten regulatory authorities have a common set of core functions: setting standards for registrants, quality assuring courses of higher education, keeping the register, and managing allegations that registrants are unfit to practise. Despite the common set of activities, there are differences in legislation, standards, approach, and efficiency reflecting the way that the councils have evolved over many years. Some of the councils regulate single professions, while the Health and Care Professions Council (HCPC) regulates 15 different professions; some regulate hundreds of thousands of registrants while some only regulate a few thousand; some have been in existence for a long time while others were founded much more recently. Most are UK wide bodies except for the regulators of pharmacy and Social Work England which regulates social workers in England only.

The ten statutory regulators are overseen by the Professional Standards Authority for Health and Social Care (the Authority) that conducts and publishes regular performance reviews for each of the regulators. The Authority also reviews all final hearing decisions in fitness to practise cases and can take action where it believes the decision is not sufficient to protect the public. The Authority shares good practice in the sector, conducts research, and promotes new ideas such as *right-touch regulation* [72].

Determining scope of practice is complex and multi-faceted in the UK with many influences. As well as the professional regulators, there are many other organizations in the UK responsible for regulating different aspects of health systems and services. Professional regulators are just one of a somewhat crowded landscape governing practice, and individual scopes of practice are influenced by local or institutional factors. Since the core focus of professional regulators in the UK is ensuring fitness to practise, a crucial challenge is ensuring there are governance and oversight arrangements to mitigate the risk to the public when individual professionals practice outside their scope of competence.

Among the 10 professional regulators in the UK, there is no common approach to determining scope of practice, nor is there any agreed definition of scope of practice. One commonality is that regulators routinely state in their standards of practice that registrants are responsible for recognizing the limits of their knowledge, skill, and experience, and must not practice unless they are capable of doing so safely and effectively. Where they provide more detailed guidance on the limits of practice, they do so in different ways.

The HCPC, for example, defines scope of practice for registrants as “the limit of your knowledge, skills and experience...Determining what is and is not part of your scope of practice will be for you to decide using your professional judgement.” [73]. In the standards of conduct, performance and ethics, the HCPC prescribes that registrants must refer a service user to another practitioner if the services needed are outside a practitioner’s scope [74]. The HCPC publishes standards of proficiency guidance [75] for each of the 15 professions that it regulates, and notes that while these standards of proficiency may inform a registrant’s scope of practice, job descriptions, employer policies, legal restrictions, coverage by professional indemnity insurance, and guidance from professional bodies will also inform scope of practice. The HCPC thus prompts practitioners to determine their scope of practice based on their professional judgment.

The General Dental Council (GDC) takes a more formalized approach, publishing a document specifically addressing scope of practice for all of its registrant groups (dental nurses, orthodontic therapists, dental hygienists, dental therapists, dental technicians, clinical dental technicians, and dentists). Unlike the HCPC’s approach, which focuses on specific knowledge areas, the GDC sets out the tasks that each group can undertake. Establishing first, like the other regulators, that scope of practice describes the areas in which registrants have the knowledge, skills and experience to practise safely and effectively, for each group it then distinguishes between the skills and abilities all registrants in the group should have, additional skills which could be developed with further training, and additional skills that can be carried out if prescribed or directed from another registrant [76]. The guidance also defines what certain groups do *not* do; dental hygienists, for example, “do not: restore teeth, carry out pulp treatments, adjust unrestored surfaces, extract teeth.” ([76], p. 7). For registrants working at the highest level of risk—dentists—the guidance is at a higher level of abstraction rather than a list of tasks are in and out of scope: dentists may “diagnose disease…prescribe and provide endodontic treatment on adult teeth…prescribe and provide fixed orthodontic treatment” ([76], p. 11). This may be changing, as the GDC is currently engaged in a scope of practice review to explore whether its guidance document is working as intended. The aim of this review is to “provide as much flexibility to dental professionals as possible, so they are using their own professional judgment about the provision of care” [77].

While it is clear that scope of practice will evolve over time, both at the individual and the profession level, it is not clear how the arrangements by which scope of practice is currently determined at either level in the UK influence this evolution; for example, whether they present barriers to innovative approaches to the delivery of care. Nor is it clear how these different arrangements and influences relate to different levels of risk being managed by the different professions, and whether there are areas of unmanaged risk to patient safety. These considerations will be important to consider post-pandemic when emergency arrangements such as accelerated licensure for international nurses [78] and redeployment of health care professionals to areas where they are working “at the limits or beyond their normal scope of practice” are reconsidered ([79], p. 2).

Table 4 details the SWOT analysis of the UK case.

**Table 4:** The SWOT analysis of the UK case

**Strengths** Transparent and publicly accountable risk-based processes with separate oversight body A range of approaches for nimble scope of practice changes	**Weaknesses** Regulators operate under different legislative frameworks Lack of a risk assessment methodology to align practice risk to relevant level of assurance Lack of shared objectives between professional and system regulators
**Opportunities** Focus on evidence-informed right-touch regulation Government reform to modernize professional regulation	**Threats** Lack of clear definition of scope of practice may weaken reform efforts Complex institutional arrangements make it difficult to determine if unmanaged risks to patient safety exist

### Leading practices for regulating scope of practice: a synthesized principle-based approach

From our results and the unique, context-specific SWOT analyses for each jurisdiction, we have developed this synthesis table of leading practices related to regulating scopes of practice that match specific regulatory principles (Table 5). These regulatory principles are adapted from Benton et al.’s [80] work and applied to our review of scope of practice regulation in these four jurisdictions.

**Table 5:** Principle-based leading practices for regulating scope of practice

Regulatory principle	Description related to scopes of practice	Leading practices
Definition	Clear definitions of professional scope that advance regulators’ mandate of protecting public safety	Uniform legislation and regulatory authorities operating nationally provide consistent and clear practice standards and regulatory frameworks (Australia)
Flexibility	Regulation sufficiently flexible and responsive to allow for timely innovation and optimization in scopes of practice	Umbrella frameworks that offer regulatory flexibility and loosen the restrictiveness of scopes of practice (many Canadian jurisdictions)
Accountability	Scope of practice regulation is transparent and contributes to high-quality and safe patient care	Transparent and publicly accountable risk-based processes with separate oversight body (UK)
Efficiency	Optimizing coherence, coordination, and communication while maintaining focus on public safety	Licensure compacts allowing licensure recognition and sharing of regulatory data across jurisdictions (US)
Collaboration	Legitimate stakeholder perspectives included in scope of practice consultations and definitions	Increased public engagement in regulatory processes such as community reference group (Australia)

## Discussion and conclusion

This novel comparative study enables us to better understand the content and context of health professional regulation and its impact on scopes of practice across jurisdictions. With the spotlight on the health workforce during the global public health crisis of COVID-19, whether regulatory frameworks for scopes of practice are currently serving and protecting the public is a critical consideration.

These four country contexts were chosen because they each have some similarities—being high-income, English-speaking countries with historic colonial ties—yet our analysis reveals some unique characteristics insofar as regulation of scopes of practice is concerned. Despite their similarities, there were nevertheless a number of challenges, including a lack of a common regulatory language, different political environments, different institutional arrangements and distributions of tasks, varying legislative foundations, ongoing reform efforts, and an absence of a clear methodology for comparison.

In the UK, health profession regulation is primarily a national-level responsibility and consistency in accountability and oversight is achieved through the Authority. In the Australian case, the move to a National Regulation and Accreditation Scheme with Ahpra and the National Boards for 15 health professions provides much greater consistency, mobility, and workforce coordination and planning at a national level. Health professional regulation is a subnational (province/state) responsibility in Canada and the US. While there is a role in each of these countries for nationally based certifying bodies in developing and implementing professional standards and licensure exams, the subnational variability in regulation in both of these jurisdictions was considered a weakness that may hinders efficient and effective workforce planning. The US move towards greater interjurisdictional cooperation through the interstate licensure compacts is a promising model.

There are varying degrees of influence of state or quasi state actors in the regulatory process around scopes of practice. This is most notable in the UK through the Authority and in Australia through Ahpra. Within some Canadian jurisdictions, the province of Ontario for example, a Health Professional Regulatory Advisory Committee, provides advice to the provincial government regarding evidence-based changes required in professional regulation, including scope of practice reform; it also applies a common set of rules applicable across all regulated health professions and is composed entirely of non-health professionals. An overarching oversight authority for regulated health professions that resembles the Authority is currently proposed in the Canadian province of British Columbia [60].

The COVID-19 pandemic has made clear the necessity of optimizing the workforce by ensuring all professionals are able to practice to full scope [81] and there have also been calls to ensure that scopes of practice are not unnecessarily restricted during the pandemic [82]. Maintaining public protection while ensuring access to the needed health workforce has become increasingly important during the pandemic. A joint statement by health professional regulators in the United States describes their “common duty” during COVID-19 as doing whatever is possible to ensure access to care across the country [83]. Lippert [84] adds that part of the regulatory mandate must be to ensure the most efficient and effective means of moving health care providers to where they are needed. While beyond the scope of this paper, the effect of changes to scope of practice on the physical and psychological health and safety of the health workforce should be considered in future work. Pandemic response plans in many countries have failed to explicitly address these critically important considerations [61] and post-pandemic reversals on scope of practice expansions may have adverse effects on health care professionals’ wellbeing [35].

There would be value in further study of whether a common definition of scope of practice would be possible across jurisdictions and examining in greater depth the various influences on professional scope of practice, including state versus professional power, and the role of continuing professional development, competency assessment, and revalidation processes on expanded scopes of practice. Recent reforms across jurisdictions emphasized realities of modern health care provision, including a need for mobility and team-based care. The impact of scope of practice regulation on these modern realities of practice should also be examined in greater detail. Finally, further study could also examine public involvement in health professional regulation and its impact on scope of practice reform. In most US states, there is little public involvement in professional self-regulation. In the UK, the *Health and Social Care Act 2008* [85] eliminated elected professional majorities on the governing boards of each regulatory council and public members now must make up at least half of each regulatory council. In Canada, there are moves for similar reform that would see boards of regulatory authorities achieve parity between professional and public members in some provinces and professions, while in others elected professional majorities remain as a vestige of traditional self-regulation. In Australia, Ahpra established a community reference group in 2013 meant to act as a conduit between communities and Ahpra and the National Boards. Greater public involvement in professional regulation, particularly around scopes of practice, would allow for a stronger public voice in health workforce planning and is another topic for future study across international jurisdictions.

## Data Availability

Not applicable.

## Abbreviations

GDC: General Dental Council (UK)
HCPC: Health and Care Professions Council (UK)
NP: Nurse practitioners
PA: Physician assistants
RHPA: Regulated Health Professions Act (Canada)
SWOT: Strengths, Weaknesses, Opportunities, Threats
